# Sickle Cell Beta-Plus Thalassemia with Subcapsular Hematoma of the Spleen

**DOI:** 10.1155/2017/3819457

**Published:** 2017-12-14

**Authors:** Suyash Dahal, Sumit Dahal, Dipesh K. C. Ghimire, Ebad Ur Rahman, Eliza Sharma

**Affiliations:** ^1^KIST Medical College and Teaching Hospital, Lalitpur, Nepal; ^2^Interfaith Medical Center, Brooklyn, NY, USA; ^3^Maimonides Medical Center, Brooklyn, NY, USA

## Abstract

While splenic complications like hypersplenism, sequestration crisis, and infarction are commonly reported in sickle cell variants like sickle cell beta-plus thalassemia, splenic rupture with hematoma is rare. We present a case of a 32-year-old young male who presented with dull left upper quadrant pain who was found to have multiple subcapsular splenic lacerations and hematoma on abdominal imaging. Hemoglobin electrophoresis confirmed sickle cell beta-plus thalassemia in the patient. There was no history of trauma, and rest of the workup for possible cause of spontaneous rupture of spleen was negative. With the patient refusing splenectomy, he was managed conservatively. Clinicians need to be aware of this rare complication of sickle cell variants.

## 1. Introduction

Sickle cell disease (SCD) refers to a group of autosomal recessive disorders characterized by hemoglobin S variant of the beta-globin gene. It affects millions of people worldwide and is particularly most prevalent in people whose ancestors came from sub-Saharan Africa, Central America, South America, Saudi Arabia, India, and Mediterranean countries. Centers for Disease Control and Prevention estimates approximately 100,000 Americans to be affected by SCD, with occurrences of about 1 in every 365 births in African Americans and about 1 in every 16,300 births in Hispanic Americans [1].

Beta-thalassemia is an inherited hemoglobinopathy caused by beta-globin gene mutations that impair the production of one or both beta-globin chains. Depending on the degree of reduction in beta-globin synthesis, it is divided into beta-thalassemia major, beta-thalassemia intermedia, and beta-thalassemia minor. It is most prevalent in Mediterranean and Southeast Asian countries [2]. Newborn screening in California estimated its incidence to be 1.8 per 100,000 infants [3].

Sickle cell beta-thalassemia is a sickle cell variant syndrome and is characterized by the compound heterozygosity for sickle and beta-thalassemia genes. It is divided into sickle cell beta^+^ thalassemia and sickle cell beta^0^ thalassemia based on a decrease or complete absence of beta-globin synthesis respectively. The gene frequency of hemoglobin S and beta-thalassemia in African Americans are estimated to be 0.04 and 0.004, respectively [4]. Newborn screening for hemoglobinopathies in California between 1998 and 2006 found the incidence of sickle cell beta^+^ thalassemia and sickle cell beta^0^ thalassemia to be 1.4 per 100,000 infants and 0.8 per 100,000 infants respectively [3]. The symptoms in patients with sickle cell beta^+^ thalassemia are less frequent and less severe than those in patients with homozygous sickle cell disease or sickle cell beta^0^ thalassemia [5, 6]. Splenic complications like hypersplenism, sequestration crisis, and infarction have been reported frequently in patients with SCD and its variants, including in sickle cell beta-plus thalassemia [7, 8]. Splenic hematoma, however, has rarely been described before. Here, we present a case of a young man with sickle cell beta-plus thalassemia who presents with spontaneous splenic rupture with multiple subcapsular hematomas.

## 2. Case Report

Our patient was a 32-year-old male who came to the hospital complaining of dull left upper quadrant pain of 2 days duration. He denied any recent trauma. He had no fever, chills, sore throat, malaise, arthralgia, skin rash, or bleeding from the gums or any other site. The rest of the review of symptoms was negative. His past medical history was significant for an unclear sickling disorder. The patient himself reported having sickle cell trait and getting exchange transfusions as a child. His family history was significant for SCD in mother.

**Figure 1:**
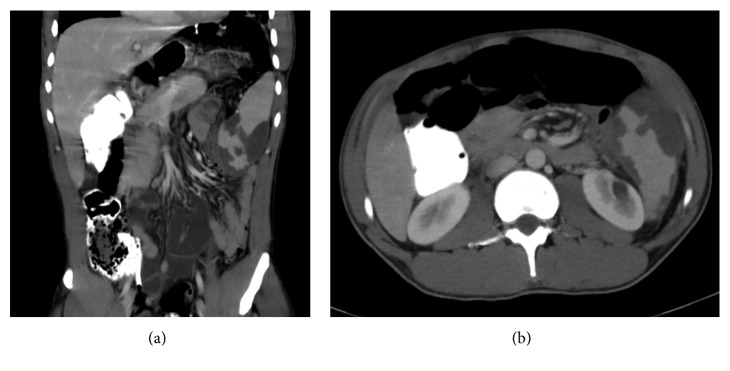
Coronal (a) and transverse (b) sections of the abdominal computed tomography scan showing multiple subcapsular hematoma in the spleen.

His vitals were stable at presentation. Physical examination was significant for splenomegaly and mild tenderness at the left upper quadrant of the abdomen with no icterus, hepatomegaly, or lymphadenopathy. Laboratory tests showed a leukocyte count of 10,400/*μ*L (normal 4,500–11,000/*μ*L), hemoglobin of 13.4 gm/dL (normal 13.5–17.5 gm/dL), hematocrit of 41% (normal 41%–53%) with mean corpuscular volume of 66.8 fL (normal 80–100 fL), platelet count of 78,000/*μ*L (normal 130,000–400,000/*μ*L), activated partial thromboplastin time of 35.7 seconds (normal 24.9–35.9 seconds), serum blood urea nitrogen of 13 mg/dL (normal 8–20 mg/dL), serum creatinine of 1 mg/dL (normal 0.4–1.3 mg/dL), total bilirubin of 2.2 mg/dL (normal 0.3–1.2 mg/dL), aspartate aminotransferase of 28 IU/L (normal 15–41 IU/L), alanine aminotransferase of 33 IU/L (normal 17–63 IU/L), and serum alkaline phosphatase of 56 IU/L (normal 32–91 IU/L). Further workup for microcytic anemia showed a reticulocyte count of 1.96% (normal 0.5%–2%), serum haptoglobin of 62 mg/dL (normal 34–200 mg/dL), serum lactate dehydrogenase of 245 IU/L (normal 98–192 IU/L), serum iron of 33 ug/dL (normal 38–169 ug/dL), iron saturation of 16% (normal 15%–55%), serum ferritin of 269 ng/mL (normal 30–400 ng/mL), and a positive sickle cell screen. Subsequent hemoglobin electrophoresis revealed 17.5% of hemoglobin A (normal 94%–98%), 7.2% of hemoglobin A2 (normal 0.7%–3.1%), 73.8% of hemoglobin S (normal 0%), and 1.5% of hemoglobin F (normal 0–2%), which was suggestive of sickle cell beta-plus thalassemia. Computed tomography (CT) scans of the abdomen showed splenomegaly with multiple splenic subcapsular lacerations and hematomas (Figure 1). No other possible cause for spontaneous splenic rupture was found in the patient. His symptomatology, physical examination, and initial lab tests were not consistent with the common causes of splenic rupture like hematological malignancies, hemophilia, or malaria. Tests for Epstein–Barr virus, cytomegalovirus, human immunodeficiency virus, and hepatitis A, B, and C viruses returned negative. So, with the diagnosis of spontaneous splenic rupture with multiple subcapsular hematomas in a patient with sickle cell beta-plus thalassemia, the patient was initially managed conservatively with bed rest, analgesia, multivitamin, folic acid, and pneumococcal and meningococcal vaccinations with plans for splenectomy going forward.

However, on the fourth day of admission, the patient started to have cough, productive of yellowish sputum and palpitation. He had low-grade fever, tachycardia, and tachypnea. Repeat CT scan of the chest showed new bilateral lower lobe infiltrates. Arterial blood gas analyses in room air showed a pH of 7.46 (normal 7.35–7.45), pCO_2_ of 37 mmHg (normal 35–45 mmHg), pO_2_ of 100 mmHg (normal 75–100 mmHg), and HCO_3_ of 26.2 mmol/L (normal 18–24 mmol/L). So, with the possible diagnosis of acute chest syndrome and/or hospital-acquired pneumonia, the patient underwent exchange transfusion with 2.8 liters of leukocyte-reduced red blood cell and broad-spectrum antibiotics. Subsequently his symptoms resolved, and his vital signs stabilized. The patient, however, refused splenectomy during his hospital stay. And with his hematological parameters stable, he was discharged home with a leukocyte count of 7,400/*μ*L, hemoglobin of 10.7 gm/dL, hematocrit of 32.5%, and platelets count of 263,000/*μ*L at discharge.

## 3. Discussion

Splenic rupture and hematoma are mostly seen with blunt abdominal trauma. However nontraumatic cases in patients with splenomegaly or underlying hematological disorders have been described [9–11]. The most common causes of spontaneous rupture of spleen include infections like Epstein–Barr virus, malaria, human immunodeficiency virus, and hepatitis virus and hematological malignancies like leukemia and lymphoma [10, 12–17]. Cases of spontaneous rupture in patients with underlying hematological disorders like hemophilia have also been reported [18, 19].

Spleen is also commonly involved in different hemoglobinopathies and usually manifest with splenomegaly, hypersplenism, acute splenic sequestration, and splenic infarction [7, 8]. Sickle cell beta-thalassemia is a sickle cell variant and is characterized by the compound heterozygosity for sickle and beta-thalassemia genes. Depending on the reduction or complete absence of beta-globin synthesis, they are divided into sickle cell beta^+^ thalassemia and sickle cell beta^0^ thalassemia. Patients with sickle cell beta-plus thalassemia have reduced synthesis of beta-globin chains rather than a complete absence and tend to have a more benign clinical course than those with sickle cell beta^0^ thalassemia or homozygous sickle cell disease [5, 20–22]. Splenic involvement occurs earlier and more frequently in patients with HbSS and sickle cell beta^0^ thalassemia as compared to sickle cell beta^+^ thalassemia [8]. There have been a few reported cases of spontaneous rupture of spleen in patients with sickle cell disease and trait [23, 24]. However, cases of sickle cell beta-plus thalassemia presenting with spontaneous splenic rupture and splenic hematoma has rarely been described before our case [25]. Mukhopadhyay et al. described a similar case of splenic hematoma, which was possibly triggered by cocaine-induced vasospasm with acute splenic infarction and subsequent hemorrhage into the infarct. Our patient, however, had no clear triggers for the hematoma. It may have resulted from spontaneous bleed into chronic splenic infarcts that our patient was predisposed to due to his underlying hemoglobinopathy. Sickled red blood cells with irregular shape and propensity to adhere to blood vessels tend to clog blood vessels and lead to congestive splenomegaly and splenic infarcts [26, 27]. This hypercoagulable state and the splenic effect may be synergized by the coexisting thalassemia [28].

In conclusion, splenic rupture and hematoma mimicking acute abdomen may rarely be the only presenting feature in patients with sickle cell disease and its variants like sickle cell beta^+^ thalassemia as seen in our case. Clinicians, therefore, need to be aware of this rare complication and consider hemoglobin electrophoresis a part of the workup for unexplained cases of nontraumatic splenic hematoma.
